# Parasites in Aphthæ

**Published:** 1842-09-03

**Authors:** 


*Parasites in Aphthæ.*—Dr. Vogel, of Munich, has observed vegetable parasites on the mucous membrane of the mouth and œsophagus of an infant a fortnight old, which died of aphthæ extending from the mouth to the cardia. These aphthæ, examined under a glass magnifying two hundred and twenty times, presented the appearance of genuine confervæ, similar to those found by Schonlein in impetigo. Some of these parasites were of a round shape, some with, some without, a central nucleus; some isolated, others grouped together; others again consisted of scattered filaments, swollen at certain points, sometimes in the middle, at others at the extremities.

All of them were colourless, and unaffected by water, ammonia, or acetic acid.—*Provin. Med. Journ.* June 25, 1842. *From Gazette de Medicale.*

